# First Necrotizing Fasciitis Caused by *Haemophilus influenza* Serotype a

**DOI:** 10.1177/2324709617736791

**Published:** 2017-10-25

**Authors:** Giang T. Quach, Jared Frisby, Kurt Kralovich, Mustafa Bohra

**Affiliations:** 1 Oakwood Southshore Medical Center, Trenton, MI, USA; 2 St. John Macomb-Oakland Hospital, Madison Heights, MI, USA; 3 Beaumont Hospital Dearborn, Dearborn, MI, USA

**Keywords:** necrotizing fasciitis, necrotizing soft tissue, *Haemophilus influenza* serotype a, *Haemophilus influenza*

## Abstract

Necrotizing fasciitis (NF) is an infrequently encountered skin infection that has high morbidity and mortality, even with prompt medical and surgical intervention. We describe the case of a 67-year-old male presenting with significant NF in his left lower extremity, despite aggressive surgical intervention, and included multiple surgical debridements, ACell Matrix, split-thickness, and negative wound VAC therapy. Ultimately, this patient required a below the knee amputation. This is the first documented case of *Haemophilus influenza* type a causing NF.

## Case Report

A 67-year-old male presented to the Beaumont Dearborn emergency department with the complaint of worsening left leg pain for 6 days. He was seen 2 days prior to this hospital visit at another facility after cutting his leg on his bed frame. He was discharged with Bactrim and Norco for presumed cellulitis. His medical history was significant for insulin-dependent type 2 diabetes mellitus, hypertension, and arthritis.

**Figure 1.**
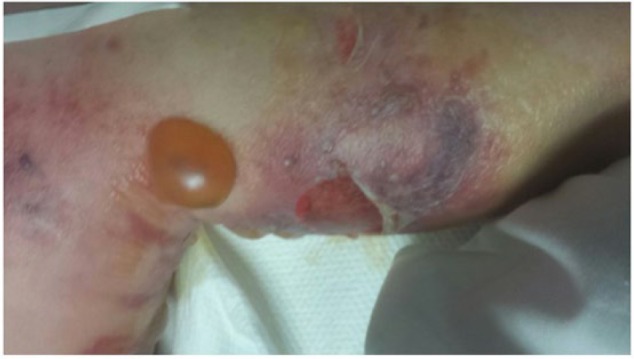
Left lower extremity on presentation; extensive necrotic tissues with multiple bullae observed from medial malleolus to proximal thigh.

There was muscle weakness and loss of motion and sensation in his left leg. Extensive bullae and erythema were found along the anterior and posterior leg, from the medial malleolus to proximal thigh (Figure 1). His vital signs were within normal limits: blood pressure 114/56, temperature 97.6, and pulse 77. Laboratory values were significant for hyponatremia (Na: 127), hypochloremia (Cl: 84), hypokalemia (2.7), hyperglycemia (Glu: 218), acute renal failure (blood urea nitrogen: 120; creatinine: 5.53; glomerular filtration rate: 10), and leukocytosis with bands (white blood cells: 23 000; bands: 8.7%).

In the emergency department, the patient was given vancomycin, clindamycin, and Zosyn. He was then brought to the operating room for emergent excisional debridement and placement of Quinton catheter for dialysis. Surgical findings included extensive necrosis of the skin, subcutaneous tissues, fascia, and underlying muscles. These findings extended from the medial thigh to the medial malleolus. Surgical debridement yielded 60 ’ 25 cm of necrotic tissue. A pulse lavage system was used to irrigate the wound and sterile dressing was applied. The patient was brought to the intensive care unit for further management, dialysis, and the plan to return for further debridement.

**Figure 2.**
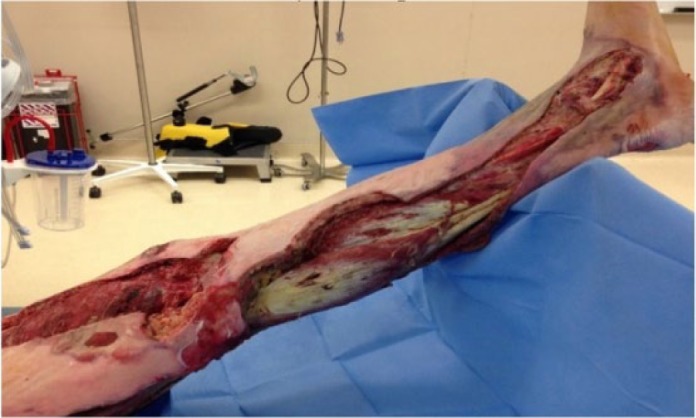
Hospital day 2: More necrotic tissues presented, especially posterior knee and beyond medial malleolus.

Patient was brought back to the operating room, hospital days 1 and 2, with continued debridement of necrotic tissue (Figure 2). Wound cultures returned showing growth of *Haemophilus influenza* serotype a that was sensitive to ceftriaxone and resistant to Bactrim. His antibiotic therapy was changed to ceftriaxone monotherapy. Blood cultures were negative for growth.

On hospital day 7, there was an application of a skin substitute to promote wound healing. After debridement of residual necrotic tissue, portions of ACell MatriStem was applied to the 30 ’ 100 cm area of exposed wounds and tendons. Sterile dressing was applied.

On hospital day 17, wound exploration was performed. Extensive necrotic and desiccated tissue was found and debrided. Wet to dry dressing was applied.

On hospital day 22, the patient had another irrigation and debridement with the intension of a split-thickness skin graft. Approximately 720 cm^2^ of skin was harvested from the right calf and thigh and applied to the left leg.

On hospital day 27, the patient returned for another wound exploration. The wound was deemed unsuitable for further skin grafting, so approximately 2000 cm^2^ of VAC negative pressure dressing was applied over the exposed wound areas.

On hospital day 30, additional debridement and replacement of the VAC negative pressure wound took place.

On hospital day 37, VAC dressing replacement and wound exploration was performed. Intraoperatively, the left knee joint was found open with synovial fluid leakage with rupture of the quadriceps and adductor tendons. Orthopedics was consulted. They agreed that a complete limb salvage or below knee amputation (BKA) was no longer an option. The patient was scheduled for an above knee amputation (AKA).

**Figure 3.**
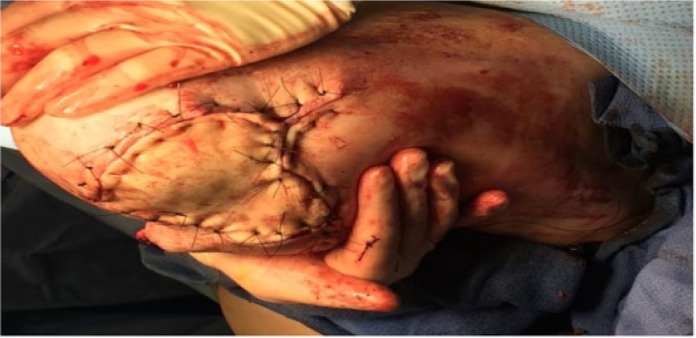
Hospital day 42: Skin and cutaneous tissue from the left distal tibia was used as a full-thickness skin to complete closure of the AKA.

On hospital day 42, the patient received a complete AKA of the left lower limb. Skin and cutaneous tissue from the left distal tibia was used as a full-thickness skin to complete closure of the AKA (Figure 3).

## Discussion

In this case, our patient presented with extensive necrotizing fasciitis (NF) of his left lower extremity that resulted in an AKA, despite aggressive interventions to salvage the limb. The decision for amputation was initially considered, but it would have required a complete hip disarticulation due to the presence of infected tissue in the proximal thigh. Additionally, a complete hip disarticulation has a greater hindrance on mobility and an increase in mortality. It was our goal to stop the spread of infection while attempting to preserve the patient’s quality of life. A BKA or AKA would have been an acceptable compromise between these goals.

With the exposure of the knee joint and tendons, we attempted to salvage the limb with ACell matrix. However, this was inhibited by desiccation and continuing tissue necrosis. Split-thickness skin grafting was then attempted. Unfortunately, poor vascularization over the areas of exposed joints and tendons likely caused the graft to fail. Subsequently, negative pressure wound VAC was placed with the hope to facilitate neovascularization and further wound healing. Wound VAC therapy, in this case, was found to be much more successful than previous methods. However, we were still unable to preserve the areas over the exposed joints and tendons. The rupture of the quadriceps and adductor tendons coupled with open knee joint and synovial fluid leakage ultimately led to no other choices but amputation.

Another remarkable feature of this case is the distinctive organism identified from the wound culture. NF is commonly caused by group A streptococci or mixed facultative/anaerobic bacteria.^1^ Our patient is a unique case where NF is caused by *H influenza* serotype a.

*Haemophilus influenza* is a gram-negative coccobacillus and a common cause of childhood upper respiratory tract infections. *H influenza* has also been known to cause meningitis, bacteremia, and pneumonia. *H influenza* are either encapsulated (typable) or unencapsulated (non-typable). Six serotypes (a-f) are known and each has capsules made from various polysaccharides. This capsule allows *H influenza* to resist phagocytosis and complement mediated lysis.^2^ Before the advent of Hib vaccination, it was the most common cause of bacterial meningitis, epiglottitis, and pneumonia in children. With the advent of Hib vaccinations, the rates of invasive Hib infections have been almost eliminated.^3^ Unencapsulated *H influenza* stains still cause upper respiratory tract infections in both children and adults, but cannot cause disseminated diseases with their lack of polysaccharide capsules.^2^

In a population study of 91 cases of invasive disease due to *H influenza*, serotype a was the most common serotype of *H influenza*. In children under the age of 5, the average incidence of Hia invasive diseases was 0.8/100 000 with the average incidence of Hib invasive diseases being 0.11/100 100 child-years. This case did not report any cases of soft-tissue infections due to *H influenza*.^4^

*H influenza* serotype f has also caused invasive infections. Of the 91 patients in a study by Urwin et al, invasive Hif infections carried a 30% mortality rate. Seventy-two percent of these patients had underlying comorbidities, such as diabetes or chronic obstructive pulmonary disease. Eighty-two percent of these 91 cases involved the upper respiratory tract. As before, no soft-tissue infections were reported in this case.^5^

There have only been 13 documented cases of NF caused by *H influenza* serotypes b, f, and e.^6,7^ This is the first documented case of *H influenza* type a causing NF, so it is unclear why one serotype was found over another. Most of the patients in previous cases of *H influenza* NF had underlying comorbidities such as immunosuppression, malignancy, and diabetes.^6,7^ In the study by Azar et al, the patient had a documented case of hypocomplementemia along with immunosuppressants for auto-immune diseases. Complement and immunoglobulin opsonization play a major role in fighting encapsulated bacteria. In particular, deficiency of C3 significantly increases the risk of *H influenza* infections. Interestingly, C5-9 deficiency is associated with increased *Neisseria* infections, not *H influenza*.^8,9^ Azar et al and Winkelstein and Moxon are the only *H influenza* NF cases that have documented hypocomplementemia, so its role is unclear in this disease process. Immunoglobulin deficiency is known to a wide variety of infections, including *H influenza*. But again, it is unknown if there is a high incidence of *H influenza* NF in patients with immunoglobulin deficiency.^7^
